# Time series analysis (2015-2022): dengue risk scenario in Goiás, Brazil

**DOI:** 10.1590/1980-549720260010

**Published:** 2026-04-10

**Authors:** Ronaldo Rodrigues de Oliveira, Ana Carolina Figueiredo Modesto, Leandro do Prado Assunção, Lindomar José Pena, Valéria Christina de Rezende Feres

**Affiliations:** I Universidade Federal de Goiás, School of Pharmacy - Goiânia (GO), Brazil.; II Universidade Federal de Goiás, Institute of Biological Sciences - Goiânia (GO), Brazil.; III Fundação Oswaldo Cruz, Aggeu Magalhães Institute - Recife (PE), Brazil.

**Keywords:** Dengue, Epidemiological models, Time series, Forecasting, Epidemiology

## Abstract

**Objective:**

To analyze confirmed cases of dengue in Goiás, Brazil between 2015 and 2022 and to estimate the risk of new outbreaks until 2026.

**Methods:**

A time series study using data from the Notifiable Diseases Information System (SINAN) was conducted. Monthly records of cases confirmed by laboratory or clinical-epidemiological criteria were included. The Seasonal Autoregressive Integrated Moving Average (SARIMA) model was applied using the R software (v.4.2.1). Stationarity, trend, seasonality, residual autocorrelation, and model fit were evaluated, with estimates obtained by maximum likelihood and 95% confidence intervals.

**Results:**

During the study period, 709,270 confirmed cases were recorded. Epidemics occurred cyclically every two years, with peaks in 2015 (101,261), 2016 (82,077), 2018 (70,794), 2019 (107,589), and 2022 (189,998), interspersed with years of lower incidence such as 2017 and the COVID-19 pandemic years (2020-2021). Serotype replacement was observed preceding major outbreaks. The SARIMA model showed good fit (Akaike Information Criterion - 1768.9; Bayesian Information Criterion - 1786.8) and predicted new peaks in 2025 (177,775 cases) and 2026 (224,100 cases).

**Conclusion:**

Dengue in Goiás displayed recurrent epidemic cycles, pointing to an increase in cases and reinforcing the need for integrated strategies based on prevention and control. The SARIMA model proved useful for surveillance and public health planning, although its accuracy may be influenced by external factors.

## INTRODUCTION

Dengue is a viral disease transmitted by mosquitoes, mainly by the vector *Aedes aegypti* ^*1*^ *.* Dengue virus (DENV 1-4) has spread to several countries as a result of the susceptibility of the human population and the wide dispersion of the vector mosquito. It is estimated that about 390 million people are annually infected with dengue, and only about a quarter of them clinically manifest the disease^2^. Historically, Brazil has the highest number of cases recorded during epidemics in the Americas. The endemic-epidemic pattern, mainly determined by the introduction and circulation of the four viral serotypes (DENV 1-4) and the spread of *Aedes aegypti* throughout the country, led to a growing increase in the incidence of cases^3^. From 1986 to 2015, Brazil recorded 11,084,755 cases of dengue, and the Midwest region represented 18% of all deaths related to DENV infection^4^.

In 2016, dengue was classified as the third neglected tropical disease (NTD) due to the impact of the disease on the population since the 1990s^5^ ^,^ ^6^. In the years 2016, 2019, 2021, and 2022, epidemics recorded more than two million cases annually, and accounted for 13,331,443 cases in the period^7^. The co-circulation of the four viral serotypes, detected in all Brazilian regions, with alternation in the predominant serotype, is a determining factor for maintaining the high number of infected cases. Among the Brazilian regions most affected by dengue, the Midwest region has presented the highest rates of dengue incidence and, until then, 2022 recorded the highest incidence, with 2,043.7 cases per 100 thousand/inhab.^8^ ^,^ ^9^ ^,^ ^10^ ^,^ ^11^.

Subsequently, in 2024, Brazil faced the largest epidemic on record, with over 10 million probable cases, incidence of 4,842.51 cases per 100 thousand/inhab., 6,321 deaths, and about six million laboratory-confirmed cases^12^. The epidemic exceeded the projection of an average of 1.9 million cases made by studies conducted with statistical modeling based on time series^13^ and in five-to-six times the records of cases with warning signs and severe cases as well as the number of deaths. The temperature rise, rainfall variations, and climatic phenomena influenced the surge in cases, reflecting the change in the life cycle of the mosquito as well as viral replication and transmission^14^.

In the state of Goiás, dengue epidemics have occurred since 1994, with the introduction of DENV-1, followed by DENV-2 in 1998, DENV-3 in 2003^15^ ^,^ ^16^, and DENV-4 in 2011^17^, reproducing the national scenario. From 2010 to 2014, 472,186 probable cases were recorded in Goiás, which resulted in 367 deaths. Since 2015, the dynamics of circulation of the four serotypes have resulted in an increase in cases with each replacement of the predominant serotype. Between 2015 and 2022, the cumulative total of 999,842 dengue cases was recorded, a period in which, for the first time, the DENV-2 has predominantly circulated, being replaced by DENV-1 in 2022, when there was a dramatic increase of 239% in the number of cases compared to the previous year^8^ ^,^ ^9^ ^,^ ^10^ ^,^ ^11^. A new historical record of dengue occurred in 2024, with 413,408 notified cases and 309,343 confirmed cases, with municipalities in the state exceeding the incidence of 2,747 per 100 thousand/inhab.^13^.

Within this context, investigations that allow to understand the occurrence of dengue, its dynamics of viral circulation, and the present and future impacts of the disease on Goiás are imperative, as there is still a shortage of regional research capable of subsidizing decisions in public health. Time series analyses have been used to identify trends, patterns of incidence and mortality, in addition to seasonality of the disease^18^. Statistical models applied to epidemiology, such as the Seasonal Autoregressive Integrated Moving Average (SARIMA), have been used in modeling temporal and seasonal patterns, in addition to forecasting epidemics, with potential application to health monitoring and planning^19^.

Thus, in this study, we aim to analyze the time series of confirmed dengue cases in Goiás between 2015 and 2022, describe their epidemiological characteristics and trends, and apply the SARIMA model to estimate the risk of new outbreaks by 2026.

## METHODS

This is a time series study with analysis of monthly dengue records in Goiás from 2015 to 2022, which used SARIMA as a statistical model for estimating cases for 2024 and new cases for the years 2025 and 2026. The state of Goiás is located in the Midwest region of Brazil, occupies an area of 340,086 km^2^, has 246 municipalities and 7.5 million inhabitants^20^. Records of notification of cases were obtained from the Notifiable Diseases Information System (*Sistema de Informação de Agravos de Notificação* - SINAN) by accessing the public database of the Department of Informatics of the Brazilian Unified Health System (DATASUS)^21^.

The analysis covered complete data on the registration of the largest and most significant dengue epidemics in Goiás between 2015 and 2022, based on the criteria for classification of dengue established by the World Health Organization (WHO) in 2009, adopted in Brazil as of 2014, and on the years of co-circulation of the four serotypes of the dengue virus. The monthly records of cases with infection confirmed by laboratory or clinical-epidemiological criteria of dengue were evaluated. At the data extraction date, data on 2023 were incomplete, and as per 2024 data, weekly cases were on the rise. These data were not included in the analysis. The inconsistency brought by these data could increase the variability in the prediction estimates of the model. In addition, the presence of incomplete data could affect the patterns of stationarity, trend, and seasonality.

Briefly, data processing was based on the creation of a dataframe^22^ named DENV based on the original databases available from SINAN and stratified per year (DENGBR15.dbc; DENGBR16.dbc; DENGBR17.dbc; DENGBR18.dbc; DENGBR19.dbc; DENGBR20.dbc; DENGBR21.dbc; DENGBR22.dbc). Data on dengue cases confirmed by clinical, laboratory, and/or epidemiological criteria were included in the DENV. Furthermore, data were grouped into dengue, dengue with warning signs, severe dengue, or discarded cases, being considered as outcome the recovery or death from dengue.

For the time series analysis, data were grouped by month and year and evaluated for: stationarity, by using the Dickey-Fuller test; trends, by the Mann-Kendall test; seasonality, by the Kruskal-Wallis test; and autocorrelation of residuals, by the Box-Pierce test. Data were modeled according to SARIMA.

Overall, the computational equation is represented as SARIMA (p, d, q)(P, D, Q), where p is the number of autoregressive orders, d is the number of non-seasonal differences, q is the number of moving average orders, P is the seasonal autoregressive order, D is the seasonal differencing order, and Q is the seasonal moving average order. The parameters of the SARIMA model were estimated using the maximum likelihood method and were calculated by point and interval statistical inference, with a 95% confidence interval (95%CI).

For the selection of the best models, the autocorrelation function (ACF) and partial autocorrelation function (PACF) of the residuals were used and the Akaike Information Criteria (AIC) and Bayesian Information Criteria (BIC) were used to compare and select the best model and calculation of estimates. All analyses were performed in spreadsheets with Microsoft Excel 2020 and with the aid of the R software, version 4.3.2.

## Data availability statement:

The dataset that supports the results of this study is available from DATASUS in an official, open, and free repository. The steps of the methodology for data processing are described in previous publication by the authors (https://doi.org/10.17504/protocols.io.j8nlko61dv5r/v1).

## RESULTS

**Figure 1.**
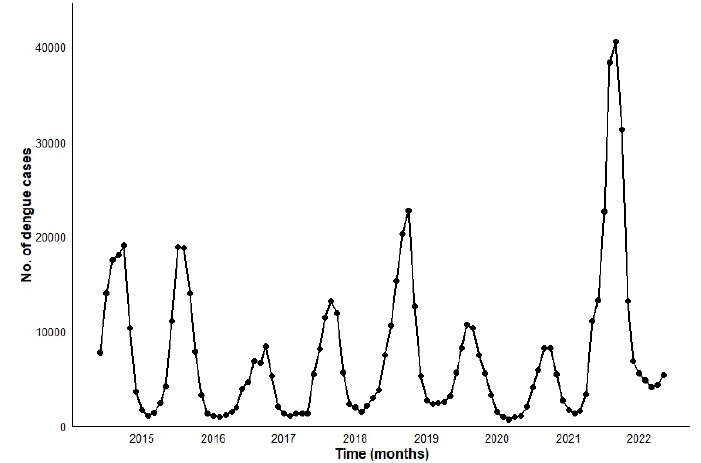
Time series of dengue cases in Goiás from 2015 to 2022. Cyclic epidemic periods every two years, with replacement of DENV-1/DENV-2/DENV-1 serotypes, preceding the largest epidemics.

The time series analysis covered 709,270 confirmed cases of dengue between 2015 and 2022 (Figure 1). We observed epidemic years with cyclic periods every two years, with peaks between 70 and 189 thousand cases in 2015 (101,261: 14.28%), 2016 (82,077; 11.57%), 2018 (70,794; 9.98%), 2019 (107,589: 15.17%), and 2022 (189,998; 26.79%), interspersed by the year 2017 (44,499; 6.27%) and the years of the COVID-19 pandemic, 2020 (56,775; 8.00%) and 2021 (56,277; 7.93%) (Figure 1). The replacement of the predominant serotype, DENV-1, for DENV-2 (2016/2017), occurred in 2016/2017, preceding the epidemics of 2018 and 2019, and again the DENV-2 was replaced with DENV-1 in 2020 and 2021, marking the anticipation of the onset of the 2022 epidemic.

According to the hypothesis tests of Dickey-Fuller (p=0.010), Mann-Kendall (p=0.484), Kruskal-Wallis (p=0.000), and Box-Pierce (p=0.653), the series is stationary, without trend, with seasonality, and residuals with no autocorrelation. Based on the results of these tests, the series were modeled with the SARIMA model, and the model with the best FAC and FACP values and the lowest values of AIC (1,768.863) and BIC (1,786.813) was chosen. The multiplicative SARIMA model with the parameters (p: 0, d: 1, q: 1)(P: 1, D: 1, Q: 3) was the most fit (Table 1).

**Table 1.** Parameters of the Seasonal Autoregressive Integrated Moving Average by age group, estimated by maximum likelihood.

Age group (years)	SARIMA*	AIC	BIC	Statistical test	p-value^†,‡,§,//^
0-9	(0,1,2)(2,1,3)	1,290.088	1,313.167	Dickey-Fuller	0.010
				Mann-Kendall	0.079
				Kruskal-Wallis	0.000
				Box-Pierce	0.996
10-19	(0,1,3)(3,1,3)	1,403.171	1,428.815	Dickey-Fuller	0.010
				Mann-Kendall	0.942
				Kruskal-Wallis	0.000
				Box-Pierce	0.463
20-29	(0,1,1)(1,1,3)	1,450.105	1,468.055	Dickey-Fuller	0.010
				Mann-Kendall	0.543
				Kruskal-Wallis	0.000
				Box-Pierce	0.517
30-39	(0,1,1)(1,1,3)	1,429.412	1,447.362	Dickey-Fuller	0.010
				Mann-Kendall	0.697
				Kruskal-Wallis	0.000
				Box-Pierce	0.508
40-49	(0,1,1)(1,1,3)	1,404.614	1,420.000	Dickey-Fuller	0.010
				Mann-Kendall	0.423
				Kruskal-Wallis	0.000
				Box-Pierce	0.954
50-59	(0,1,2)(1,1,3)	1,372.779	1,390.729	Dickey-Fuller	0.010
				Mann-Kendall	0.478
				Kruskal-Wallis	0.000
				Box-Pierce	0.864
60-69	(0,0,3)(3,0,0)	1,293.124	1,311.074	Dickey-Fuller	0.010
				Mann-Kendall	0.482
				Kruskal-Wallis	0.000
				Box-Pierce	0.807
70-79	(0,0,3)(3,0,0)	1,175.009	1,195.524	Dickey-Fuller	0.010
				Mann-Kendall	0.271
				Kruskal-Wallis	0.000
				Box-Pierce	0.709
80-89	(0,0,3)(3,0,0)	961.397	981.912	Dickey-Fuller	0.010
				Mann-Kendall	0.142
				Kruskal-Wallis	0.000
				Box-Pierce	0.911
90-100	(0,1,1)(3,1,1)	682.687	700.638	Dickey-Fuller	0.010
				Mann-Kendall	0.077
				Kruskal-Wallis	0.000
				Box-Pierce	0.429

SARIMA: Seasonal Autoregressive Integrated Moving Average; AIC: Akaike Information Criterion; BIC: Bayesian Information Criterion; *SARIMA Model; ^†^Dickey-Fuller Test; ^‡^Mann-Kendall Test; ^§^Kruskal-Wallis Test; ^//^Box-Pierce Test.

**Figure 2.**
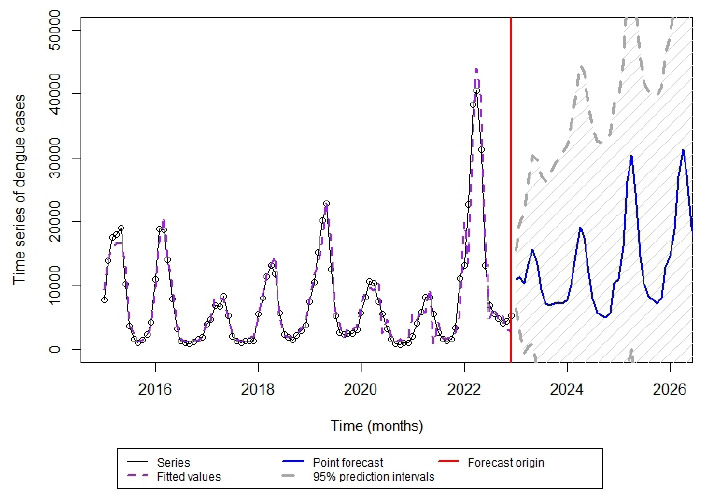
Time series of dengue cases with the SARIMA model (0,1,1) x (1,1,3) and estimation of the number of dengue cases in 2023, 2024, and prediction for 2025 and 2026.

Based on the multiplicative SARIMA model (0,1,1) x (1,1,3), estimates for dengue cases in the years 2025 and 2026 were 177,775 (95%CI 11,047-1,475,150) and 224,100 (95%CI 6,906-2,350,817), respectively (Figure 2). Estimates covered the years 2023 (120,318; 95%CI 50,762-343,032) and 2024 (121,510; 95%CI 19,883-827,096), and for 2025 preliminary results have been obtained until Epidemiological Week 35; in this sense, the prediction is relevant for 2026.

**Figure 3.**
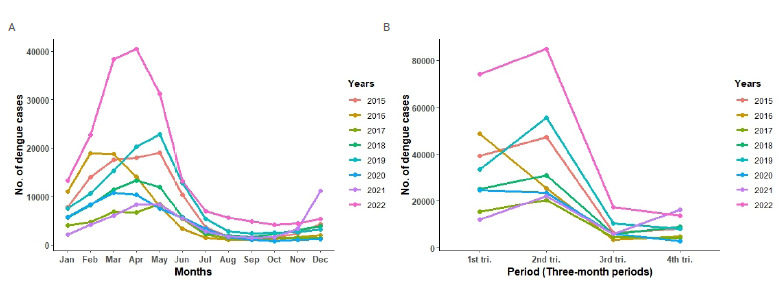
Number of dengue cases over the years (A) and per trimester (B).

The seasonality identified by the Kruskal-Wallis test is observed in the first trimesters of 2015 to 2022 (Figure 3). Overall, dengue cases begin in January, with an exponential increase in the months of February, March, and April, demonstrating that the first trimester of each year represents the period of greatest dengue burden for the health services and health surveillance actions. However, in some years, specifically 2015, 2017, and 2019, there was late exponential growth, with maximum peaks of dengue cases occurring after the first trimester (Figure 3).

A: Number of dengue cases reported per month over the years; B: Number of dengue cases reported per trimester; 1^st^ tri: First trimester; 2^nd^ tri: Second trimester; 3^rd^ tri: Third trimester; 4^th^ tri: Fourth trimester.

**Figure 4.**
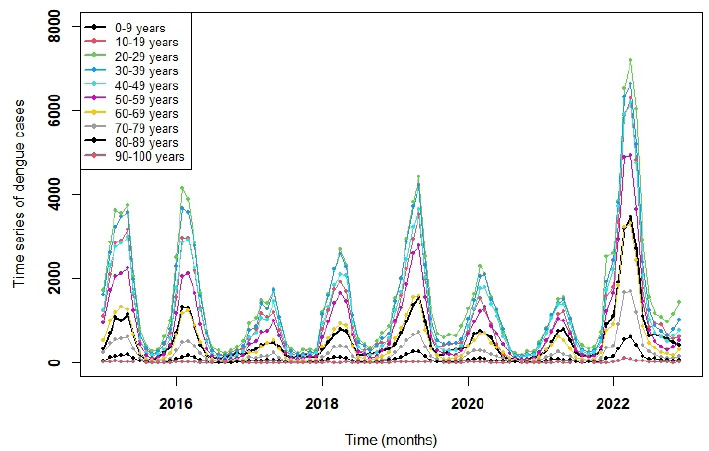
Number of dengue cases over the years distributed by age group.

**Figure 5.**
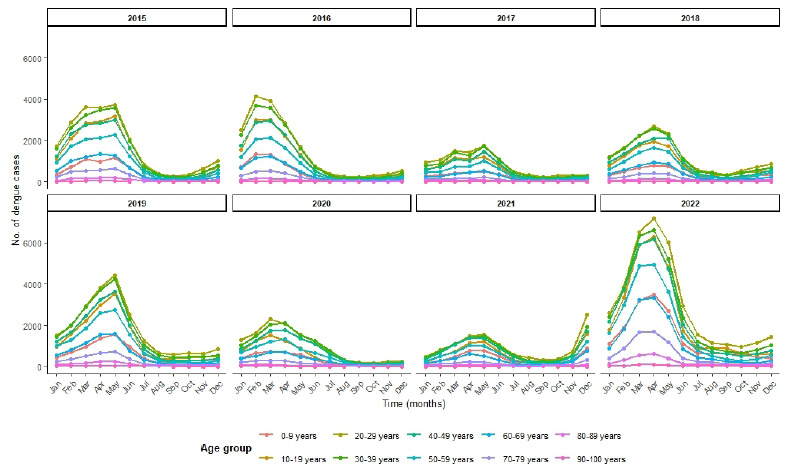
Number of dengue cases throughout the months of each year separated by age group. Anticipation of dengue cases in 2021 for the 2022 epidemic.

Data were stratified by age groups, ranging from 0-9 years to 90-100 years, and time series were grouped to identify patterns and trends (Figure 4). We observed that the age groups 20-29 years (n=144,941) and 30-39 years (n=131,600) accounted for the highest number of cases. Overall, the data showed a similar age distribution throughout the study period. In most years, dengue incidence exponentially increased during the first trimester, with a substantially higher number of cases in 2022. Notably, an atypical rise in dengue cases was detected in November and December 2021, preceding the major increase observed in the first trimester of 2022 (Figure 5).

In addition, in all data separated by age group, the Dickey-Fuller, Mann-Kendall, Kruskal-Wallis, and Box-Pierce tests were employed to verify stationarity, trend, seasonality, and autocorrelation of the residuals. The detailed values of the hypothesis tests are provided in Table 1, and it is worth highlighting that data on all age groups presented stationarity and seasonality. Conversely, we observed no trends or autocorrelation of residuals in the data.

**Figure 6.**
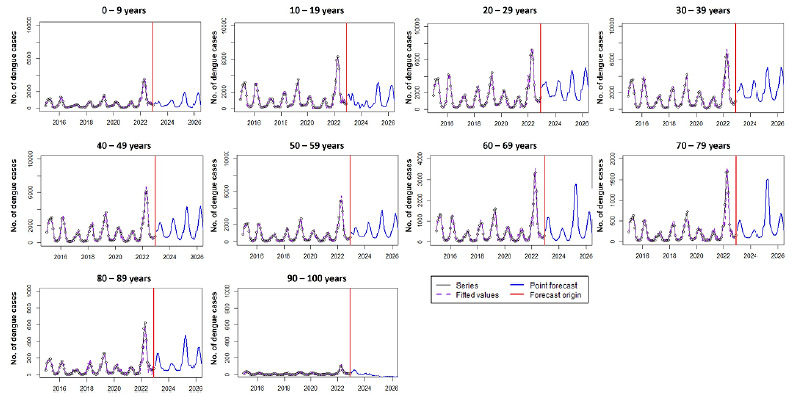
Prediction of the number of dengue cases for the years 2025 and 2026 by age group. Increase in cases from the age group over 60 years.

The age groups with the highest estimates of new cases are between 20 and 29 years (28,950; 4.08% in 2025 and 32,916; 4.64% in 2026) and between 30 and 39 years (30,191; 4.26% in 2025 and 34,595; 4.88% in 2026). Among older adults, we observed 12,153 cases in 2025 (1.71%) and 6,760 in 2026 (0.95%) in the age group from 60 to 69 years; 6,971 in 2025 (0.98%) and 3,926 in 2026 (0.55%) in the age group between 70 and 79 years; and 2,418 in 2025 (0.34%) and 1,973 in 2026 (0.28%) in the age group between 80 and 89 years (Figure 6).

## DISCUSSION

The analysis of the time series of dengue included the years of the largest epidemics ever recorded in Goiás, totaling 709,270 confirmed cases. The years 2015, 2016, 2018, 2019, and 2022 presented peak records between 70 and 189 thousand cases, especially 2022, which represented a major epidemic, even after a sequence of years with high concentration of cases. We verified an epidemic pattern, with cycles every two years, after alternation in the circulation of the predominant serotype, DENV-1/DENV-2/DENV-1, preceding the years of the largest epidemics, 2019 and 2022 (Figure 1).

Based on the SARIMA modeling, the time series presented an increasing estimate of cases for 2025 (177,775; 95%CI 11,047-1,475,150) and, especially, for 2026 (224,100; 95%CI 6,906-2,350,817), which may reflect a worse epidemiological scenario for dengue. Considering the official numbers of Goiás in 2025, up to the Epidemiological Week 35^20^, 136,904 cases were reported, figures close to what was predicted by the model. In addition, when carrying out the series with data from 2015 to 2022, estimates for 2023 (120,318; 95%CI 50,762-343,032) and 2024 (121,510; 95%CI 19,883-827,096) could be compared to actual notifications, which totaled 95 and 413 thousand cases^23^, respectively.

In 2024, the epidemiological scenario of dengue was unusual, determined by climate change that impacted viral transmission and resulted in a historical epidemic in Brazil^13^. The El Niño phenomenon, already notified by the WHO^24^, contributed to this scenario and caused the predictive models elaborated by InfoDengue/Fiocruz, even in the worst projected scenario, to be overcome by successive weeks of increased cases. By the end of April, the country has already accumulated more than four million probable cases of dengue^13^.

The influence of the El Niño phenomenon^24^ in 2023 and 2024 caused changes in Brazil, such as increase in temperature and rainfall, as happened in previous years (2010 to 2019)^25^. These changes may favor the decrease in the time of infection and emergence of the vector mosquito and, therefore, increase the population density and infectivity of mosquitoes for viral transmission^26^.

In Goiás, this context was reflected in the increase in the number of cases, characterizing one of the largest epidemics recorded in the historical series, with almost double the cases compared to 2022, in addition to the rise in serious cases and deaths and the reemergence of DENV-3^23^. The estimates of this analysis did not reflect what was expected for 2024 due to intervention of strong climate determinants. However, when considering the confidence interval of the 2024 estimate (121,510; 95%CI 19,883-827,096), the observed number of 413 thousand reported cases is encompassed by the estimated range. The influence of this external factor suggests that the increase in cases projected for 2025 in the present study was anticipated to 2024, given that the high magnitude of transmission led to an earlier peak of cases in the country^13^. Nonetheless, the occurrence of epidemics has multifactorial aspects, ranging from the effectiveness of entomological control actions to the described aspects, such as susceptibility of the population to infection by different serotypes^27^, environmental conditions and basic sanitation^28^, in addition to globalization and dissemination of the virus and vector mosquitoes^29^.

In the analysis of the historical series, 2021 represented the foreshadowing of the 2022 epidemic by the early increase in the case registration curve in November and December, contrasting with previous years (Figure 3). Researchers suggest that the increase in the number of cases usually occurs during periods of greater rainfall^30^ ^,^ ^31^ ^,^ ^32^. These variations, when monitored in due time, may indicate a change in the expected pattern, as observed with the anticipation of rainfall, increase in temperature, and consequent substantial growth in cases as of January 2024.

Another relevant aspect that may have influenced the 2022 epidemic was the advent of the COVID-19 pandemic, both due to the probable underreporting of cases in 2020 and 2021 and to the reduction of vector control actions due to priority mobilization against the coronavirus^33^. Conversely, the circulation dynamics of dengue serotypes involved successive replacements of the predominant serotype, as occurred in 2017, when DENV-2 was reintroducted and replaced DENV-1, and again DENV-1 became the prevalent serotype in 2021^34^. In 2024, DENV-2 became the predominant serotype, accounting for 74.4% of the serotypes detected, which highlights the dynamics of circulation of serotypes and the recording of people susceptible to infection, leading to the emergence of new infected cases.

In this study, the estimate of new cases for 2025 and 2026 for Goiás warns of new epidemic peaks, with a high incidence of cases. We emphasize that the current scenario of maintenance of high-risk areas for case incidence^35^ and the imminent reintroduction with possible sustained circulation of the DENV-3 serotype constitute an additional risk for the intensification of cases, with direct impact on morbidity and mortality and burden of the healthcare network. In this context, it is worth noting the historical series of dengue in Brazil, especially the period of 2002/2003, when the emergence of DENV-3 resulted in a significant increase in the number of cases and the occurrence of more severe clinical forms of the disease^21^.

The profile of individuals infected with dengue in Brazil are young adults^36^. According to the stratification by age group, there is a predominance of cases in young adults (20-39 years), which concentrate a greater absolute number of infections and, therefore, have a central role in the maintenance of transmission. However, the number of cases estimated for the age group of over 60 years is concerning. Although these individuals are a smaller contingent among the cases, they are considered vulnerable to dengue virus infection because its association with comorbidities increases the risk of complications and death^35^. The estimates of cases in older adults for 2025 and 2026 accounted for 12.14 and 5.65%, respectively, and represent an alert for the identification of cases and adequate management in this group. According to official data on 2025, there was an increase in cases with warning signs and severe dengue, corresponding to an increasing lethality in those aged 65 to 79 years (4.9%) and 80 years or over (14.1%)^8^ ^,^ ^9^ ^,^ ^10^ ^,^ ^11^.

Our results show that the multiplicative SARIMA model with the parameters (p: 0, d: 1, q: 1)(P: 1, D: 1, Q: 3) was appropriately fit to the time series of dengue in Goiás. It presented a comprehensive analysis, with consistency in historical data and maintenance of the seasonal temporal pattern of dengue epidemics, with an estimated increase in the number of cases in 2025 and 2026. Authors of previous studies used time series analysis and applied the SARIMA model for predicting cases and impending outbreaks of dengue^37^ and other infectious diseases with seasonal patterns, consisting in a useful analysis and forecasting tool for epidemiological surveillance^38^.

The complexity of dengue epidemics reflects the regional scenario that, in Goiás, is configured by the concomitant circulation of DENV-1 and DENV-2 in the largest municipalities. The co-circulation of the four serotypes in other regions of the state, where the climate is tropical seasonal with rainy summer, at the beginning of each year, and dry winter, as of June^31^, is consistent with the seasonality pattern observed in this study, characteristic of the Midwest region. Actions for vector control in municipalities may vary, and the maintenance of high-risk areas for infestation and incidence of cases is observed^23^, in addition to the co-circulation of chikungunya and Zika virus^35^ ^,^ ^39^.

As limitations of the study, we mention the no inclusion of climate data in the model and the use of notification data by SINAN, which may vary in terms of quality in different stages of identification, investigation, and completeness of variables, according to the technical-operational conditions of the regional epidemiological surveillance system. Furthermore, the accuracy of estimates can be influenced by external variables, such as climatic conditions, and large-scale interventions such as vaccination.

The simple and accurate methodology used, based on confirmed cases of dengue, and the application of replicable modeling systems resulted in analyses imperative to evaluate the temporal trend of the incidence of the disease, in a scenario not yet influenced by the introduction of dengue vaccines, allowing a robust baseline for future comparisons.

Thus, we consider that, although the number of cases has fluctuated in recent years, these still evidence an alarming scenario of high transmission, infected people and deaths, representing a considerable impact on health care. Our data, especially with regard to the forecast for 2026, raise an alert, considering that after the spread of DENV-3 in Brazil and the record of autochthonous cases in the state of Goiás, the scenario constitutes as a risk for the reversal of the predominant serotype, in accordance with the criteria established by the Special Operations Committee for the implementation of actions in response to dengue epidemics^13^.

Thus, the results of the analysis reinforce the need for integrated and sustained strategies of prevention and control in the face of the increasing burden of the disease at the regional level. The use of predictive models, based on updated data, is a relevant tool for epidemiological surveillance and public health planning.
