# Supplementary material for: Time series analysis (2015-2022): dengue risk scenario in Goiás, Brazil
Source: Rev Bras Epidemiol. 2026 Apr 10;29:e260010. doi: 10.1590/1980-549720260010 (PMC13078831; doi:10.1590/1980-549720260010)
Supplement: Translation

# Translation: Análise de série temporal (2015-2022): cenário de risco para dengue em Goiás

**DOI:** 10.1590/1980-549720260010.2

**Authors:** Ronaldo Rodrigues de Oliveira, Ana Carolina Figueiredo Modesto, Leandro do Prado Assunção, Lindomar José Pena, Valéria Christina de Rezende Feres

**Affiliations:** I Universidade Federal de Goiás, Faculdade de Farmácia - Goiânia (GO), Brasil.; II Universidade Federal de Goiás, Instituto de Ciências Biológicas - Goiânia (GO), Brasil.; III Fundação Oswaldo Cruz, Instituto Aggeu Magalhães - Recife (PE), Brasil.

**Keywords:** Dengue, Modelos epidemiológicos, Série temporal, Modelos de predição, Epidemiologia

## Abstract

**Objetivo:**

Analisar os casos confirmados de dengue em Goiás entre 2015 e 2022 e estimar o risco de novos surtos até 2026.

**Métodos:**

Estudo de série temporal com dados do Sistema de Informação de Agravos de Notificação (SINAN). Foram incluídos registros mensais de casos confirmados por critérios laboratoriais ou clínico-epidemiológicos. O modelo Autorregressivo Integrado de Médias Móveis Sazonais (SARIMA) foi aplicado utilizando o *software* R (v.4.2.1). Foram avaliados estacionariedade, tendência, sazonalidade, autocorrelação dos resíduos e ajuste do modelo, com estimativas por máxima verossimilhança e intervalo de confiança de 95%.

**Resultados:**

No período foram registrados 709.270 casos confirmados. As epidemias ocorreram de forma cíclica a cada dois anos, com picos em 2015 (101.261), 2016 (82.077), 2018 (70.794), 2019 (107.589) e 2022 (189.998), intercalados por anos de menor incidência, como 2017 e os anos de pandemia da COVID-19 (2020-2021). Observou-se substituição de sorotipos antecedendo os maiores surtos. O modelo SARIMA apresentou bom ajuste (Critério de Informação de Akaike - AIC 1.768,9; Critério de Informação Bayesiano - BIC 1.786,8) e previu novos picos em 2025 (177.775 casos) e 2026 (224.100 casos).

**Conclusão:**

A dengue em Goiás apresenta ciclos epidêmicos recorrentes, que apontam para a intensificação de casos e reforçam a necessidade de estratégias integradas e sustentadas de prevenção e controle. O modelo SARIMA mostrou-se útil para vigilância e planejamento em saúde pública, embora sua precisão possa ser influenciada por fatores externos.

## INTRODUÇÃO

A dengue é uma doença viral transmitida por mosquitos, principalmente pelo vetor *Aedes aegypti* ^*1*^. O vírus da dengue (DENV 1-4) espalhou-se por vários países em consequência da suscetibilidade da população humana e da ampla dispersão do mosquito vetor. Estima-se que aproximadamente 390 milhões de pessoas sejam infectadas com dengue anualmente, e apenas cerca de um quarto delas manifesta a doença clinicamente^2^. Historicamente, o Brasil detém o maior número de casos registrados durante as epidemias ocorridas nas Américas. O padrão endemoepidêmico, determinado principalmente pela introdução e circulação dos quatro sorotipos virais (DENV 1-4) e dispersão do *Aedes aegypti* pelo país, levou ao aumento crescente na incidência de casos^3^. De 1986 e 2015, o Brasil apresentou 11.084.755 casos de dengue, e a Região Centro-Oeste representou 18% de todas as mortes relacionadas à infecção pelos DENV^4^.

Em 2016, a dengue foi classificada como a terceira doença tropical negligenciada (DTN) em razão do impacto da doença na população desde os anos 1990^5^ ^,^ ^6^. Nos anos de 2016, 2019, 2021 e 2022, as epidemias registraram mais de 2 milhões de casos anualmente, e no período atingiram o patamar de 13.331.443 de casos^7^. A cocirculação dos quatro sorotipos virais, detectados em todas as regiões brasileiras, com alternância no sorotipo predominante, constitui um fator determinante para a manutenção do alto número de casos infectados. Entre as regiões brasileiras mais afetadas pela dengue, a Região Centro-Oeste tem apresentado as maiores taxas de incidência de dengue, e em 2022 atingiu 2.043,7 casos por 100 mil/hab.^8^ ^,^ ^9^ ^,^ ^10^ ^,^ ^11^.

Em 2024, o Brasil enfrentou a maior epidemia de todos os tempos, com registro de mais de 10 milhões de casos prováveis, incidência de 4.842,51 casos por 100 mil hab., 6.321 óbitos e quase 6 milhões de casos confirmados laboratorialmente^12^. A epidemia superou a projeção de, em média, 1,9 milhão de casos feita por estudos com modelagem estatística baseada em séries temporais^13^ e em 5-6 vezes os registros de casos com sinais de alarme e casos graves, assim como no número de óbitos. A elevação das temperaturas, as variações de precipitação e fenômenos climáticos influenciaram a explosão de casos, refletindo-se na alteração no ciclo de vida do mosquito, assim como na replicação viral e transmissão^14^.

No estado de Goiás, epidemias de dengue têm ocorrido desde 1994, com a introdução do DENV-1, seguido pelo DENV-2 em 1998, DENV-3 em 2003^15^ ^,^ ^16^ e DENV-4 em 2011^17^, reproduzindo o cenário nacional. De 2010 a 2014, Goiás registrou 472.186 casos prováveis, que resultaram em 367 mortes. Desde 2015 a dinâmica de circulação dos quatro sorotipos tem produzido o aumento de casos a cada substituição do sorotipo predominante. Entre 2015 e 2022, o total acumulado de 999.842 casos de dengue foi registrado, período em que pela primeira vez o DENV-2 circulou predominantemente, sendo substituído pelo DENV-1 em 2022, ano em que houve aumento dramático de 239% no número de casos em comparação com o ano anterior^8^ ^,^ ^9^ ^,^ ^10^ ^,^ ^11^. Novo registro histórico de dengue ocorreu em 2024, com 413.408 casos notificados e 309.343 casos confirmados, com municípios no estado superando a incidência de 2.747 por 100 mil hab.^13^.

Nesse contexto, investigações que permitam compreender a ocorrência da dengue, sua dinâmica de circulação viral e os impactos presentes e futuros da doença em Goiás são fundamentais, uma vez que ainda há escassez de pesquisas regionais capazes de subsidiar decisões em saúde pública. As análises de séries temporais vêm sendo utilizadas para identificar tendências, padrões de incidência e mortalidade, bem como a sazonalidade da doença^18^. Modelos estatísticos aplicados à epidemiologia, como o Autorregressivo Integrado de Médias Móveis Sazonais (SARIMA), têm sido empregados na modelagem de padrões temporais e sazonais, além da previsão de epidemias, com potencial aplicação no monitoramento e planejamento em saúde^19^.

Assim, este estudo propõe-se a analisar a série temporal de casos confirmados de dengue em Goiás entre 2015 e 2022, descrever suas características epidemiológicas e tendências e aplicar o modelo SARIMA para estimar o risco de novos surtos até 2026.

## MÉTODOS

Este é um estudo de série temporal com análise dos registros mensais de dengue em Goiás dos anos de 2015 a 2022, que utilizou o SARIMA como modelo estatístico para a estimativa de casos para 2024 e novos casos para os anos de 2025 e 2026. O estado de Goiás está localizado na Região Centro-Oeste do Brasil, ocupa área de 340.086 km^2^, possui 246 municípios e 7,5 milhões de habitantes^20^. Os registros de notificação de casos foram obtidos no Sistema de Informação de Agravos de Notificação (SINAN) pelo acesso à base de dados pública DataSUS^21^.

A análise abrangeu os dados completos do registro das maiores e mais significativas epidemias de dengue em Goiás entre 2015 e 2022, com base nos critérios de classificação da dengue estabelecidos pela Organização Mundial da Saúde (OMS) em 2009, adotados no Brasil a partir de 2014, e nos anos de cocirculação dos quatro sorotipos do vírus da dengue. Avaliaram-se os registros mensais de casos com infecção confirmada por critérios laboratoriais ou clínico-epidemiológicos de dengue. Na data de extração dos bancos de dados, os dados de 2023 estavam incompletos, e os dados de 2024 em pleno aumento de casos semanais. Esses dados não foram incluídos na análise. A inconsistência que eles trariam poderia aumentar a variabilidade nas estimativas de predição do modelo. Além disso, a presença de dados incompletos poderia afetar os padrões de estacionariedade, tendência e sazonalidade.

Brevemente, o tratamento do banco de dados baseou-se na construção de um *dataframe* ^*22*^ nomeado como DENV com base nos bancos de dados originais disponíveis no SINAN e estratificados por ano (DENGBR15.dbc; DENGBR16.dbc; DENGBR17.dbc; DENGBR18.dbc; DENGBR19.dbc; DENGBR20.dbc; DENGBR21.dbc; DENGBR22.dbc). Foram incluídos no DENV dados de casos com dengue confirmada por critério clínico, laboratorial e/ou epidemiológico. Ainda no *dataframe* DENV, os dados foram agrupados em dengue, dengue com sinais de alarme, dengue grave ou casos descartados, sendo considerada como desfecho a recuperação ou o óbito por dengue.

Para a análise das séries temporais, os dados foram agrupados por mês e ano e avaliados quanto à estacionariedade utilizando o teste de Dickey-Fuller, quanto às tendências por meio do teste de Mann-Kendall, quanto à sazonalidade com o teste de Kruskal-Wallis e quanto à autocorrelação dos resíduos com o teste de Box-Pierce. Os dados foram modelados de acordo com o SARIMA.

Em geral, a equação computacional é representada como SARIMA (p, d, q)(P, D, Q), em que p é o número de parâmetros autorregressivos, d é o número de diferenciações para que a série se torne estacionária, q é o número de parâmetros de médias móveis, P é a ordem do termo autorregressivo sazonal, D é o número de diferenciações sazonais e Q é a ordem do termo de média móvel sazonal. Os parâmetros do modelo SARIMA foram estimados utilizando o método de máxima verossimilhança e calculados por meio de inferência estatística pontual e intervalar, com intervalo de confiança de 95% (IC95%).

Para a seleção dos melhores modelos, foram utilizadas as funções de autocorrelação (FAC) e autocorrelação parcial (FACP) dos resíduos e os Critérios de Informação de Akaike (AIC) e Bayesiano (BIC) para comparar e selecionar o melhor modelo e cálculo das estimativas. Todas as análises foram realizadas em planilhas eletrônicas com Microsoft Excel 2020 e com o auxílio do *software* R, versão 4.3.2.

## Declaração de disponibilidade de dados:

Dados disponíveis (O conjunto de dados que dá suporte aos resultados deste estudo está disponível pelo DataSUS em repositório oficial, aberto e de acesso gratuito. As etapas da metodologia para tratamento dos dados estão descritas na publicação dos autores (https://doi.org/10.17504/protocols.io.j8nlko61dv5r/v1).

## RESULTADOS

A análise da série temporal abrangeu 709.270 casos confirmados de dengue entre 2015 e 2022 (Figura 1). Observaram-se anos epidêmicos com períodos cíclicos a cada dois anos, com registros de picos entre 70 e 189 mil casos em 2015 (101.261: 14,28%), 2016 (82.077; 11,57%), 2018 (70.794; 9,98%) 2019 (107.589: 15,17%) e 2022 (189.998 (26,79%), intercalados pelo ano de 2017 (44.499; 6,27%) e os anos de pandemia da COVID-19, 2020 (56.775; 8,00%) e 2021 (56.277; 7,93%) (Figura 1). A substituição do sorotipo predominante, DENV-1 para DENV-2 (2016/2017), ocorreu em 2016/2017, antecedendo as epidemias de 2018 e 2019, e novamente o DENV-2 substituiu o DENV-1 (2020/2021), marcando a antecipação do início da epidemia de 2022.

**Figura 1.** Série temporal dos casos de dengue em Goiás no período de 2015 a 2022. Períodos epidêmicos cíclicos a cada dois anos, com substituição dos sorotipos DENV-1/DENV-2/DENV-1 antecedendo as maiores epidemias.

Os testes de hipótese de Dickey-Fuller (p=0,010), Mann-Kendall (p=0,484), Kruskal-Wallis (p=0,000) e Box-Pierce (p=0,653) revelaram que a série é estacionária, sem tendência, com sazonalidade e resíduos não autocorrelacionados. Com base nos resultados desses testes, as séries foram modeladas com o modelo SARIMA, sendo o melhor modelo escolhido aquele com melhores FAC, FACP e menores valores de AIC (1.768,863) e BIC (1.786,813). O modelo SARIMA multiplicativo com os parâmetros (p: 0, d: 1, q: 1)(P: 1, D: 1, Q: 3) foi o mais adequado (Tabela 1).

**Tabela 1.** Parâmetros dos modelos Autorregressivo Integrado de Médias Móveis Sazonais (SARIMA) por faixa etária, estimados por máxima verossimilhança.

Faixa etária (anos)	SARIMA*	AIC	BIC	Teste estatístico	Valor de p^†,‡,§,//^
0-9	(0,1,2)(2,1,3)	1.290,088	1.313,167	Dickey-Fuller	0,010
				Mann-Kendall	0,079
				Kruskal-Wallis	0,000
				Box-Pierce	0,996
10-19	(0,1,3)(3,1,3)	1.403,171	1.428,815	Dickey-Fuller	0,010
				Mann-Kendall	0,942
				Kruskal-Wallis	0,000
				Box-Pierce	0,463
20-29	(0,1,1)(1,1,3)	1.450,105	1.468,055	Dickey-Fuller	0,010
				Mann-Kendall	0,543
				Kruskal-Wallis	0,000
				Box-Pierce	0,517
30-39	(0,1,1)(1,1,3)	1.429,412	1.447,362	Dickey-Fuller	0,010
				Mann-Kendall	0,697
				Kruskal-Wallis	0,000
				Box-Pierce	0,508
40-49	(0,1,1)(1,1,3)	1.404,614	1.420,000	Dickey-Fuller	0,010
				Mann-Kendall	0,423
				Kruskal-Wallis	0,000
				Box-Pierce	0,954
50-59	(0,1,2)(1,1,3)	1.372,779	1.390,729	Dickey-Fuller	0,010
				Mann-Kendall	0,478
				Kruskal-Wallis	0,000
				Box-Pierce	0,864
60-69	(0,0,3)(3,0,0)	1.293,124	1.311,074	Dickey-Fuller	0,010
				Mann-Kendall	0,482
				Kruskal-Wallis	0,000
				Box-Pierce	0,807
70-79	(0,0,3)(3,0,0)	1.175,009	1.195,524	Dickey-Fuller	0,010
				Mann-Kendall	0,271
				Kruskal-Wallis	0,000
				Box-Pierce	0,709
80-89	(0,0,3)(3,0,0)	961,397	981,912	Dickey-Fuller	0,010
				Mann-Kendall	0,142
				Kruskal-Wallis	0,000
				Box-Pierce	0,911
90-100	(0,1,1)(3,1,1)	682,687	700,638	Dickey-Fuller	0,010
				Mann-Kendall	0,077
				Kruskal-Wallis	0,000
				Box-Pierce	0,429

SARIMA: Autorregressivo Integrado de Médias Móveis Sazonais; AIC: Critério de Informação de Akaike; BIC: Critério de Informação Bayesiano.

*Modelo SARIMA; ^†^Teste de Dickey-Fuller; ^‡^Teste de Mann-Kendall; ^§^Teste de Kruskal-Wallis; ^//^Teste de Box-Pierce.

Com base no modelo SARIMA (0,1,1) x (1,1,3) multiplicativo, as estimativas para os casos de dengue nos anos de 2025 e 2026 foram de 177.775 (IC95% 11.047-1.475.150) e 224.100 (IC95% 6.906-2.350.817), respectivamente (Figura 2). As estimativas passaram pelos anos de 2023 (120.318; IC95% 50.762-343.032) e 2024 (121.510; IC95% 19.883-827.096), e para 2025 se têm resultados preliminares até a Semana Epidemiológica 35; nesse sentido, a predição faz-se pertinente para 2026.

**Figura 2.** Série temporal dos casos de dengue com o modelo SARIMA (0,1,1) x (1,1,3) e estimativa do número de casos de dengue em 2023, 2024 e predição para 2025 e 2026.

A sazonalidade identificada pelo teste de Kruskal-Wallis é observada nos primeiros trimestres dos anos de 2015 a 2022 (Figura 3). De maneira geral, os casos de dengue têm início no mês de janeiro, com aumento exponencial nos meses de fevereiro, março e abril, demonstrando que o primeiro trimestre de cada ano representa o período de maior carga de dengue para o serviço e ações de vigilância em saúde. No entanto, em alguns anos, especificamente 2015, 2017 e 2019, houve crescimento exponencial tardio, com picos máximos de casos de dengue ocorrendo após o primeiro trimestre (Figura 3).

**Figura 3.** Número de casos de dengue ao longo dos anos (A) e por trimestre (B).

Legenda: Número de casos de dengue reportados por mês ao longo dos anos (A); Número de casos de dengue reportados por trimestre (B). Qtr1: Primeiro trimestre; Qtr2: Segundo trimestre; Qtr3: Terceiro trimestre; Qtr4: Quarto trimestre.

Os dados foram estratificados por faixas etárias, variando de 0-9 anos a 90-100 anos, e foram criadas séries temporais agrupadas para identificar padrões e tendências (Figura 4). Percebe-se que a população nas faixas etárias de 20-29 (n=144.941) anos e 30-39 anos (n=131.600) concentram o maior número de casos. No geral, os dados apresentaram perfil semelhante em todos os anos do período estudado, observando-se aumento exponencial no primeiro trimestre de cada ano, com destaque para maiores frequências de casos em 2022. Observou-se um comportamento atípico nos casos de dengue nos meses de novembro e dezembro de 2021, um aumento exponencial, de forma antecipada ao incremento de casos de dengue no primeiro trimestre de 2022 (Figura 5).

**Figura 4.** Número de casos de dengue ao longo dos anos distribuído por faixa etária.

**Figura 5.** Número de casos de dengue ao longo dos meses de cada ano separados por faixa etária. Antecipação de casos de dengue em 2021 para a epidemia de 2022.

Adicionalmente, em todos os dados separados por faixa etária, foram aplicados os testes de Dickey-Fuller, Mann-Kendall, Kruskal-Wallis e Box-Pierce para verificar a estacionariedade, tendência, sazonalidade e autocorrelação dos resíduos. Os valores detalhados dos testes de hipóteses estão disponíveis na Tabela 1, sendo importante ressaltar que os dados de todas as faixas etárias apresentaram estacionariedade e sazonalidade. Em contrapartida, não foram observadas tendências nem autocorrelações dos resíduos nos dados.

As faixas etárias com as maiores estimativas de casos novos são entre 20 e 29 anos (28.950; 4,08%) em 2025 e 32.916 (4,64%) em 2026, e entre 30 e 39 anos (30.191; 4,26%) em 2025 e (34.595; 4,88%) em 2026. Entre os idosos, observam-se 12.153 casos em 2025 (1,71%) e 6.760 em 2026 (0,95%) na faixa de 60 a 69 anos; 6.971 em 2025 (0,98%) e 3.926 em 2026 (0,55%) na faixa entre 70 e 79 anos; e 2.418 em 2025 (0,34%) e 1.973 em 2026 (0,28%) na faixa entre 80 e 89 anos (Figura 6).

**Figura 6.** Previsão do número de casos de dengue para os anos 2025 e 2026 por faixa etária. Alta de casos a partir da faixa de idade acima de 60 anos.

## DISCUSSÃO

A análise da série temporal da dengue incluiu os anos de maiores epidemias já registradas em Goiás, perfazendo o total de 709.270 casos confirmados. Os anos de 2015, 2016, 2018, 2019 e 2022 apresentaram registros de picos entre 70 e 189 mil casos, com destaque para 2022, que representou a capacidade de produzir epidemias explosivas, mesmo após uma sequência de anos com elevada concentração de casos. Revelou-se um padrão epidêmico, com ciclos a cada dois anos, após alternância na circulação do sorotipo predominante, DENV-1/DENV-2/DENV-1, antecedendo os anos de maiores epidemias, 2019 e 2022 (Figura 1).

Com base na modelagem SARIMA, a série temporal apresentou estimativa crescente de casos para 2025 (177.775; IC95% 11.047-1.475.150) e, especialmente, para 2026 (224.100; IC95% 6.906-2.350.817), podendo refletir um pior cenário epidemiológico para a dengue. Considerando-se os números oficiais de Goiás em 2025, até a Semana Epidemiológica 35^20^ foram notificados 136.904 casos, números próximos ao previsto pelo modelo. Além disso, ao projetar a série com dados de 2015 a 2022, as estimativas para 2023 (120.318; IC95% 50.762-343.032) e 2024 (121.510; IC95% 19.883-827.096) puderam ser comparadas às notificações reais, que totalizaram 95 mil e 413 mil casos^23^, respectivamente.

Em 2024, o cenário epidemiológico da dengue foi anômalo, determinado por alterações climáticas que impactaram a transmissão viral e resultaram em uma epidemia histórica no Brasil^13^. O fenômeno El Niño, já sinalizado pela OMS^24^, contribuiu para esse quadro e fez com que os modelos preditivos elaborados pelo InfoDengue/Fiocruz, mesmo no pior cenário projetado, fossem superados por sucessivas semanas de aumento de casos. Até o final de abril, o país já acumulava mais de 4 milhões de casos prováveis de dengue^13^.

A influência no fenômeno El Niño^24^ em 2023 e 2024 provocou alterações como aumento na temperatura e precipitação no Brasil, assim como em anos anteriores 2010 a 2019^25^. Essas alterações podem favorecer a diminuição do tempo de infecção e de eclosão dos mosquitos vetores e, portanto, aumentar a densidade populacional e a infectividade dos mosquitos para a transmissão viral^26^.

Em Goiás, esse contexto refletiu-se no aumento do número de casos, estabelecendo um marco entre as maiores epidemias da série histórica, com quase o dobro de casos em comparação a 2022, além do crescimento de casos graves e óbitos e da reemergência do DENV-3^23^. O que se observa é que as estimativas desta análise não refletiram o esperado para 2024 por intervenção de forte determinante climático. Entretanto, ao se observar o intervalo de confiança da estimativa de 2024 (121.510; IC95% 19.883-827.096), percebe-se que o número de 413 mil casos correspondente às notificações reais estava previsto pelo modelo. A influência desse fator externo sugere que o aumento de casos projetado para 2025 no presente estudo foi antecipado para 2024, visto que a alta magnitude da transmissão levou a um pico de casos mais precoce no país^13^. Entretanto, a ocorrência de epidemias tem aspectos multifatoriais, que vão desde a efetividade das ações de controle entomológico aos aspectos descritos, tais como a suscetibilidade da população à infecção por diferentes sorotipos^27^, condições ambientais e de saneamento básico^28^, além da globalização e disseminação viral e de mosquitos vetores^29^.

Na análise da série histórica, o ano de 2021 representou o prenúncio da epidemia de 2022 pela elevação antecipada na curva de registro de casos, nos meses de novembro e dezembro, contrastando com anos anteriores (Figura 3). Estudos sugerem que o aumento no número de casos geralmente ocorre durante períodos de maior precipitação de chuvas^30^ ^,^ ^31^ ^,^ ^32^. Essas variações, quando monitoradas oportunamente, podem sinalizar uma mudança no padrão esperado, tal como observado com a antecipação das chuvas, aumento da temperatura e consequente explosão dos casos a partir de janeiro de 2024.

Outro aspecto relevante que pode ter influenciado a epidemia de 2022 foi o advento da pandemia de COVID-19, tanto pela provável subnotificação dos casos em 2020 e 2021 quanto pela redução das ações de controle vetorial devida à mobilização prioritária contra o coronavírus^33^. Por outro lado, a dinâmica de circulação dos sorotipos da dengue envolveu sucessivas substituições do sorotipo predominante, tal como ocorreu em 2017, ano com reintrodução do DENV-2 em substituição do DENV-1, e novamente o DENV-1 tornou-se o sorotipo prevalente em 2021^34^. Em 2024, o DENV-2 voltou a ser o sorotipo predominante, com 74,4% dos sorotipos detectados, o que ressalta a dinâmica de circulação de sorotipos e o encontro de pessoas suscetíveis à infecção, levando à emergência de novos casos infectados.

Neste estudo, a estimativa de novos casos para os anos de 2025 e 2026 para Goiás alertam para novos picos epidêmicos, com alta incidência de casos. Ressalta-se que o cenário atual de manutenção de áreas de alto risco de incidência de casos^35^ e a iminente reintrodução com possível circulação sustentada do sorotipo DENV-3 configuram um risco adicional para a intensificação de casos, com impacto direto na morbimortalidade e na sobrecarga da rede de atenção à saúde. Nesse contexto, destaca-se a relevância de observar a série histórica de dengue no Brasil, especialmente o período de 2002/2003, quando a emergência do DENV-3 resultou em expressivo aumento do número de casos e na ocorrência de formas clínicas mais graves da doença^21^.

O perfil de indivíduos infectados pela dengue no Brasil são os adultos jovens^36^. A estratificação por faixa etária revelou predominância de casos em adultos jovens (20-39 anos), que concentram maior número absoluto de infecções e, portanto, têm papel central na manutenção da transmissão. Contudo, é preocupante o número de casos estimados para a faixa de idade de acima de 60 anos. Apesar de esses indivíduos serem um contingente menor dentre os casos pela doença, eles são considerados vulneráveis à infecção pelo vírus da dengue porque a associação com comorbidades eleva o risco de complicações e óbito^35^. As estimativas de casos em idosos para 2025 e 2026 somaram 12,14 e 5,65%, respectivamente, e representam um alerta para a identificação de casos e o manejo adequado neste grupo. Os dados oficiais de 2025 mostram aumento de casos com sinais e alarmes e casos graves, correspondendo a uma letalidade crescente naqueles com faixa etária de 65 a 79 anos (4,9%) e 80 anos ou mais (14,1%)^8^ ^,^ ^9^ ^,^ ^10^ ^,^ ^11^.

Nossos resultados mostram que o modelo SARIMA multiplicativo com os parâmetros (p: 0, d: 1, q: 1)(P: 1, D: 1, Q: 3) se ajustou adequadamente à série temporal de dengue em Goiás. Apresentou uma análise abrangente, com consistência nos dados históricos e manutenção do padrão temporal sazonal das epidemias de dengue, com estimativa de aumento no número de casos nos anos de 2025 e 2026. Estudos anteriores utilizaram a análise de séries temporais e a aplicação do modelo SARIMA para a predição de casos e surtos iminentes dengue^37^ e outras doenças infecciosas que exibem padrões sazonais, sendo uma ferramenta de análise e previsão útil para a vigilância epidemiológica^38^.

A complexidade das epidemias de dengue reflete o cenário regional que, em Goiás, se configura pela circulação concomitante do DENV-1 e DENV-2 nos maiores municípios. A cocirculação dos quatro sorotipos em outras regiões do estado, onde o clima é tropical sazonal com verão chuvoso, no início de cada ano e inverno seco, a partir de junho^31^, condiz com o padrão de sazonalidade observado neste estudo, característico da Região Centro-Oeste. As ações para o controle vetorial nos municípios podem variar e observa-se a manutenção de áreas de alto risco de infestação e incidência de casos^23^ e, ainda, a cocirculação de chikungunya e Zika^35^ ^,^ ^39^.

Como limitações do estudo, têm-se a não inclusão de dados climáticos no modelo e o uso de dados provenientes de notificação pelo SINAN, que podem sofrer variabilidade na qualidade em diferentes etapas da identificação, investigação e completude de variáveis, conforme as condições técnico-operacionais do sistema de vigilância epidemiológica regional. Ressalta-se, ainda, que a acurácia das estimativas pode ser influenciada por variáveis externas, como condições climáticas e intervenções de larga escala, como a vacinação.

A metodologia simples e precisa utilizada, baseada em casos confirmados de dengue, e a aplicação de sistemas de modelagem replicáveis resultaram em análises fundamentais para avaliar a tendência temporal da incidência da doença, em um cenário ainda não influenciado pela introdução de vacinas contra a dengue, permitindo uma linha de base robusta para futuros comparativos.

Desta forma, consideramos que, mesmo com a oscilação no número de casos entre os anos recentes, estes ainda demonstram um cenário alarmante de alta transmissão, de pessoas infectadas e óbitos, representando alto impacto para a assistência em saúde. Nossos dados, especialmente no que se refere à previsão para 2026, servem de alerta, pois após a disseminação do DENV-3 no Brasil e o encontro de casos autóctones no estado de Goiás, o cenário constitui-se como de risco para a inversão de sorotipo predominante, em conformidade com os critérios estabelecidos pela Comissão de Operações Especiais para a ativação de ações em resposta às epidemias por dengue^13^.

Assim, os resultados da análise reforçam a necessidade de estratégias integradas e sustentadas de prevenção e controle perante a crescente carga da doença em nível regional. O uso de modelos preditivos, baseados em dados atualizados, configura-se como ferramenta relevante para a vigilância epidemiológica e o planejamento em saúde pública.
